# Synergistic effects of exogenous IAA and melatonin on seed priming and physiological biochemistry of three desert plants in saline-alkali soil

**DOI:** 10.1080/15592324.2024.2379695

**Published:** 2024-07-29

**Authors:** Youwei Zhang, Lei Wang, Xuebo Li, Hao Wen, Xiao Yu, Yixuan Wang

**Affiliations:** Forestry College, Inner Mongolia Agricultural University, Hohhot, China

**Keywords:** Desert plants, IAA, melatonin, saline soil, seed germination, osmoregulation

## Abstract

To investigate the synergistic effect of IAA and melatonin (MT) on three plants to alleviate the effects of salt damage on plants, we aim to determine the optimal concentrations of exogenous hormone treatments that improve salinity resistance for each species. In this experiment, three desert plants, *Sarcozygium xanthoxylon*, *Nitraria tangutorum*, and *Ammopiptanthus mongolicus*, which are common in Wuhai City, were used as plant materials. Two time periods (12 h,24 h) of exogenous hormone IAA (100 μmol/L) and exogenous melatonin concentration (0, 100, 200, 300 μmol/L) were used to treat the three desert plants in saline soil under different conditions of exogenous IAA and exogenous melatonin. The results indicate that under different concentrations of exogenous IAA and melatonin, the germination rate and vigor of the three desert plant species in saline-alkaline soil improved. However, as the concentration of melatonin increased, the germination rate and vigor of these desert plants were inhibited. Whereas, plant height, root length, leaf length, fresh weight, dry weight, and root vigor of the three desert plants were alleviated under different conditions of exogenous IAA and exogenous melatonin. under the action of two exogenous hormones, the low concentration of melatonin decreased their malondialdehyde content and increased their proline content. As melatonin levels increased, the activity of antioxidant enzymes also rose initially, followed by a subsequent decline. This study highlights the synergistic effects of two exogenous hormones on the critical role of cell osmomodulators and antioxidant enzyme activity in combating salinity damage in three desert plants.

## Introduction

Due to increasing soil salinity worldwide, its high salinity, low nutrient content, and poor soil structure, which are unfavorable for crop growth and development, seriously affect agricultural productivity and ecological sustainability.^1–4^ According to statistical data, many fertile soils have become salt-affected wastelands, and their problem occurs within the borders of more than 100 countries, such as China, Australia, India, Mexico, Pakistan, Syria, and the United States of America.^1^

In the research sample site, the precipitation of Wuhai City in the past years is 159.8 mm, with average precipitation and average evaporation of 3289 mm. In the past decade, underground mining in the area has intensified, leading to an increase in disposal sites. The region features various soil types, including saline soil, wind-eroded soil, gray desert soil, brown calcareous soil, and coal cinder soil. These soils are severely affected by salinization and alkalization, with significant erosion occurring. There is an urgent need for ecological restoration, and vegetation selection should focus on selecting the most resistant ones capable of implementing extensive vegetation restoration to regulate soil nutrients and PH levels. Therefore, considering the selection of appropriate vegetation, three desert plants, namely, *Sarcozygium xanthoxylon*, *Nitraria tangutorum*, and *Ammopiptanthus mongolicus*, are proposed to be selected as the pioneer plants for ecological restoration; however, it is difficult to survive in this salinized land without treatment, so it is necessary to carry out exogenous hormone treatments in a reasonable manner to improve their resistance to adapt to the local environment. The application of phytohormones, such as indole acetic acid (IAA) is of increasing interest to improve phytoremediation potential.^2^ IAA triggering can accelerate seed germination by regulating endogenous hormones and sucrose metabolism.^3^ In previous studies, growth hormone can interact with ROS critical for regulating plant growth under salt stress; indole-3-acetic acid (IAA) mediates a variety of growth and developmental responses, detoxifies ROS, and promotes cytokinesis,^4^ Exogenously supplied growth hormones promote plant growth in various plant growth in plant species.^5^ Phytohormones such as growth hormone (IAA) and photosynthesis are involved in regulating and promoting adventitious rooting.^6^ IAA also mitigates the detrimental effects of salt stress on root architecture by inducing rooting.^7^ Alae Ahmad Jabbour et al.^8^ found that 50 ppm IAA was superior to 100 ppm cytokinin (kinetin), 90% sulfuric acid and 20% acetic acid for seed initiation in the treatment of *Juniperus procera* seeds. Macias Leon Maria A et al.^9^ found that Seed germination improved with Eth (30 and 100 μm), Eth(100 μM)+IAA (10 μM), and IAA(3 uM) when onion (*Allium cepa* L.) seeds were immersed in hormone solu treaments. The synergistic effect of IAA with Eth enhanced the rate of seed germination. Jin Mengran et al.^10^ found that endogenous IAA and gibberellin (GA3) contents of *Syringa villosa* were significantly higher than that of the control after IAA treatment, while abscisic acid (ABA) content was significantly decreased. Liu Chang et al.^11^ showed that exogenous IAA significantly promoted mesocotyl elongation in MS24B (short mesocotyl autocotyledonous line) by increasing cell length. In MS24B, IAA treatment increased amylase activity to degrade starch into soluble sugars and hexokinase activity was increased to consume the increased soluble sugars to provide more energy. This energy will contribute to increased PM H-ATPase activity and expression of extended protein-related genes, which will ultimately promote acidification of the plasma membrane for cell elongation in MS24B.

Melatonin (N-acetyl-5-methoxytryptamine) is an indole derivative of tryptophan, first discovered in the pineal gland of cattle. Melatonin (MT) is a widely studied biomolecule with a dual function as an antioxidant and signaling molecule. To counteract salinity, plants activate internal tolerance mechanisms, mainly through osmoregulation and enhancement of compatible solutes, such as proline and glycine betaine.^12^ Recent research advances have led to a deeper understanding of melatonin, explaining its role in enhancing seed germination, regulating plant flowering time, promoting plant growth, and acting as a critical regulator under biotic and abiotic stress conditions,^13, 14,15^ In addition, in the study of Zhao Yongteng et al.^16^ it was found that MT induced complex changes, including up-regulation of gene expression, regulation of signal transduction, regulation of hormone levels and enhancement of the antioxidant system, altered the metabolic pathways and response mechanisms under environmental stress, and promoted the synthesis of metabolites. Hussain Sadiq et al.^17^ found that primordial dormancy of seeds of annual saline plants *Z. simplex* and *P. oleracea* was attenuated by priming with different concentrations of MT. Liu Yumo et al.^18^ found that MT significantly shortened the time for seeds to break through the seed coat and improved stress-inhibited root growth. α-Amylase and β-Amylase gene expression, antioxidant enzymes, and amylase activities were increased by MT. The starch and soluble sugar contents were changed accordingly. Wang Jiajie et al.^19^ showed that MT application significantly reduced the negative effects of salt stress on wheat seed germination. The oxidative load was reduced by inducing high activity of antioxidant enzymes. Wu Si-Qi et al.^20^ found that soybean seeds had higher SOD levels in both S13 and S30 when 100 μmol/L MLT was added on days 5, 6, and 7 of germination, which MLT application significantly increased the total phenol content of soybean sprouts collected on days 6 and 7, and that 100 μmol/L and 300 μmol/L MLT treatments resulted in genes associated with antioxidant properties in soybean sprouts higher up-regulation

*Sarcozygium xanthoxylon*, *Tribulus terrestris* family (Zygophyllaceae), ultra-arid small shrubs, is a genus of plants endemic to the desert regions of central Asia, widely distributed in deserts, steppe-enveloped deserts, and desertified steppe zones, and is also ecologically important.^21^ *Nitraria tangutorum*, Nitrariaceae, Nitraria is a multi-branched ecologically resilient and pulp-economical shrub of Tribulus Terrestris (Zygophyllaceae), which is mainly distributed in the deserts and saline-alkaline lands of Xinjiang, Qinghai, Ningxia, Gansu and Inner Mongolia in China, and shows vital salt and drought tolerance.^22^ *Ammopiptanthus mongolicus*, Fabaceae, with strong resistance to adversity, is an excellent shrub and rare and endangered plant in desert areas that mainly prevents wind and fixes sand and plays an essential role in the maintenance of desert ecosystems and the greening of deserts.^23^ All three desert plants are drought-resistant, mainly distributed in western Inner Mongolia, Ningxia, and Gansu, and can adapt to the natural environment of the Western Ordos region. They are all excellent water-retaining plants.

The conditions in the mining area of Wuhai City are much harsher, and the salinization of mining soils can cause varying degrees of damage to desert plants. There are fewer previous studies on the salinity tolerance of the three desert plants, and so far, it is not clear how the synergistic effect of exogenous IAA and exogenous melatonin will mitigate the harmful effects of saline soils on these three desert plants. This study aimed to analyze and discuss the germination rate, germination potential, seedling biomass accumulation, and physiological mechanisms of three desert plant species in saline-alkaline soils using two exogenous hormones. The goal is to provide a theoretical basis for cultivation in saline-alkaline soils and to promote the development and utilization of saline-tolerant species.

## Materials and methods

Test materials and experimental design: (research sample site and soil sampling site) Local representative soil in Wuhai City was selected as the substrate of the soil (saline soil), and the soil sampling site was the Guangna Mining Area in Wuhai City, the depth of deep soil sampling was 20 cm, and the microelements and physicochemical properties of the soil were determined. Seeds of three desert plants were soaked with IAA at 100 μmol/L, and two groups of soaking times were set up, soaking for 12 h and soaking for 24 h. The melatonin (MT) treatment groups were CK, T1, T2, and T3, and the melatonin concentrations were 0, 100, 200, and 300 μmol/L, respectively.

Test grouping:

### Indicators and methods of measurement

In Table 1,the experiment was carried out in the greenhouse of Mengenhua in Haibowan District, Wuhai City, where newly picked and whole ripe, similarly grown seeds with no dried particles were used, sterilized with 0.5% K_2_Mno_4_ for 8–10 min and then rinsed with deionized water for 3–5 times. To promote the number of germination, seeds were treated with 98% concentrated sulfuric acid for 10 min. The seeds were spotted in cavity trays, and 96 seeds were planted in each group, with three replications for each treatment group. The soil was sample soil, and the planting depth was 2.5 cm. After planting, the soil was watered once with the melatonin solution, with the concentration set for each subgroup. 3d after germination, it was watered once more, and the foliage was sprayed with melatonin solution with the corresponding concentration on the 7th, 15th, and 25th day. The germination of the three desert plants was recorded daily, and on the 14th day, 10 seedlings were selected from each treatment to measure the fresh weight and calculate the germination rate and germination potential.

**Table 1.** Experimental design of three desert plants.

Duration of IAA-soaked seeds	Treatment group	Seeds soaked with different concentrations of melatonin and subsequent spraying on seedling leaves. (μmol/L)	Type of soil substrate
100μmol/LIAA immersion for 12h	CK (12)	0	Saline soil
	T1 (12)	100	Saline soil
	T2 (12)	200	Saline soil
	T3 (12)	300	Saline soil
100μmol/LIAA immersion for 24h	CK (24)	0	Saline soil
	T1 (24)	100	Saline soil
	T2 (24)	200	Saline soil
	T3 (24)	300	Saline soil

Note: In the following, CK(12) denotes a 12-hour IAA immersion with a melatonin concentration of 0; T1(12) denotes a 12-hour IAA immersion with a melatonin concentration of 100 μmol/L; T2(12) denotes a 12-hour IAA immersion with a melatonin concentration of 200 μmol/L; T3(12) denotes a 12-hour IAA immersion with a melatonin concentration of CK(24) represents IAA immersion for 12 hours with a melatonin concentration of 0; T1(24) represents IAA immersion for 12 hours with a melatonin concentration of 100 μmol/L; T2(24) represents IAA immersion for 12 hours with a melatonin concentration of 200 μmol/L; T3(24) represents IAA immersion for 12 hours with a melatonin concentration of 300 μmol/L; and T3(24) represents IAA immersion for 12 hours with a melatonin concentration of 300 μmol/L. was 300 μmol/L.

Germination was determined by using seed breakthrough as the criterion for germination; germination was determined by using (total number of seeds germinated before harvest/number of seeds tested) × 100%; Germination potential was determined using (total number of seeds germinated within 5 d/number of seeds supplied) × 100%; Ten seedlings were taken after 21 d for the determination of morphological indicators.^24^ Fresh weight: weighed accurately using a one-thousandth electronic balance. Dry weight: single seedlings from each group were killed in an oven at 105°C for 30 min and then transferred to 80°C for drying to constant weight, and the dry weight of roots and seedlings was accurately weighed using a one-thousandth balance and recorded. Plant height, root length, and leaf length were measured accurately using vernier calipers and repeated three times for each group.

### Seedlings were taken at 30 d to determine various physiological and biochemical indices

#### Determination of physiological indicators in seedlings of three desert plants

Determination of plant osmoregulatory substances: soluble sugars and soluble proteins were determined by anthrone colorimetry and by Caumas Brilliant Blue (G-250) staining;^25,26^ The thiobarbituric acid method^22^ determined malondialdehyde content, proline content was determined by the acid ninhydrin colorimetric method,^27^ and relative conductivity was determined by the conductivity meter method.^28^ Chlorophyll a, b, carotenoids, and total chlorophyll content were determined by ethanol, acetone immersion method.^29^ Root vigor: Root vigor was determined by TTC method;^30^ Antioxidant enzyme activities: superoxide dismutase (SOD) activity was determined by NBT photochemical reduction method,^31^ peroxidase (POD) activity was determined by guaiacol colorimetric assay;^32^ catalase (CAT) activity was determined by potassium permanganate titration method.^33^

Data processing: Microsoft Excel 2019, SPSS 27.0, and Origin 2021 software were used to organize and analyze the data and make graphs, and LSD and Duncan method were used for multiple comparisons of the significance of the differences (*p* < 0.05), and the data of each index were expressed as the “Mean±Standard Error” of 3 repetitions. The data of each index were expressed as the “Mean±Standard Error” of 3 replications.

Table 2 shows the Physico-chemical properties of post-sampling saline soils determined before the experiment

**Table 2.** Measurements of various physicochemical properties in saline soils used by three desert plants.

Index	PH	water content/%	Soil bulk density/ (g·cm^3^	Total porosity/%	Salt content/%	Urease/ (u·g-^1^)	Catalase/ (u·g-^1^)	Organic matter/%	Alkaline hydrolyzable nitrogen/ (mg/kg)	Available potassium/ (mg/kg)	Available phosphorus/ (mg/kg)
	*8.580*	9.000	17.131	0.625	5.978	0.165	0.015	4.800	42	12.025	14.1

Physico-chemical properties of post-sampling saline soils determined prior to the experiment

## Result

### Effect of different treatment times with IAA immersion and different concentrations of melatonin MT on seed germination of three desert plants in saline soil

**Figure 1.**
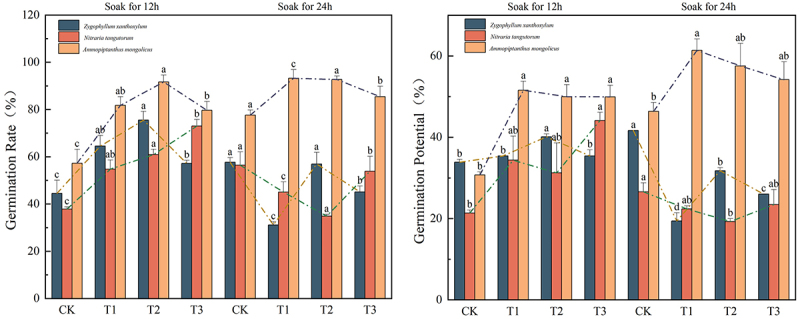
Effect of IAA immersion and exogenous melatonin treatment at different times on germination potential and germination percentage of three desert plants.

In Figure 1, the addition of different concentrations of melatonin significantly enhanced the germination and germination potential of the three desert plants in the treatment groups as compared to the CK group, with the highest peak germination potential of 73.05% in the T3(12) group of the overlord (*Sarcozygium xanthoxylon*) seeds, which was 69.59% higher as compared to the CK(12) group, and 69.10% higher in the CK(24) group of the overlord (*Sarcozygium xanthoxylon*) seeds CK(24) group had the highest germination potential of 41.64%, which was 23.10% higher as compared to CK(12) group. The germination percentage and germination potential of white spurge (*Nitraria tangutorum*) seeds after the T3(12) group reached the highest peak of 73.05% and 44.13%, respectively. Its germination percentage and germination potential increased by 92.95%, 106.70%, 29.37%, and 66.09% compared to CK(12) and CK(24) groups, respectively, and the germination potential of white spurge (*Nitraria tangutorum*) showed no significant difference (*p* > 0.05) among the fractions of CK(24), T1(24), T2(24), and T3(24). The germination rate and germination potential of sand holly (*Ammopiptanthus mongolicus*) seeds in the T1(24) group reached the highest peak values of 93.23% and 61.37%, respectively. Its germination rate and germination potential were increased by 62.73% and 99.71% and 20.13% and 66.08% compared to the CK(12) and CK(24) groups, respectively, and there was no significant difference (*p* > 0.05) between the fractions of holly CK(24), T1(24), T2(24), and T3(24).

### Effects of IAA immersion and exogenous melatonin treatments at different times on plant height, leaf length, and root length in three desert plants

**Figure 2.**
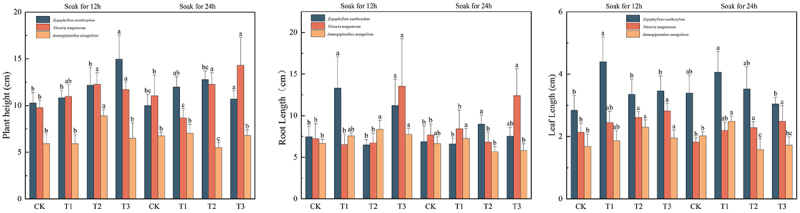
Trends of plant height, root length, and leaf length changes in three desert plants at different times of IAA immersion and exogenous melatonin treatments.

In Figure 2, the treatment groups significantly (*p* < 0.05) enhanced the plant height of bully (*Sarcozygium xanthoxylon*), white thorn (*Nitraria tangutorum*), and sand holly in the fractions treated with different times of IAA immersion and different concentrations of melatonin compared to CK(12) and CK(24), the maximum value of plant height of bully (*Sarcozygium xanthoxylon*) T3 (12) plant height had a maximum value of 13.75 cm, which was 26.85% and 37.50% higher than CK (12) and CK (24), respectively, and white thorn (*Nitraria tangutorum*) plant height of the T3 group immersed for 24 h had a maximum value of 13.47 cm, which was higher than CK (12) and CK (24), 37.59% and 21.79%, respectively. The maximum plant height of *Ammopiptanthus mongolicus* in the T1(24) group was 9.18 cm, which was 54.96% and 36.00% higher than that of the CK(12) and CK(24) groups, respectively.

The root length of *Sarcozygium xanthoxylon* after IAA soaking for 12 h showed a trend of increasing in decreasing and then increasing in all the fractions, among which the T1 group had the maximum root length of 13.32 cm, which was 78.55% and 93.89% higher than that of the CK(12) and CK(24) groups, respectively, and both of them were significantly greater than that of all the fractions soaked for 24 h (*p* < 0.05). White thorn (Nitraria tangutorum) showed a tendency to decrease and then increase in all fractions after 12 h of IAA soaking, in which the root length of the T3 group was the largest at 13.54 cm, which increased by 87.02% and 76.53% compared to CK(12) and CK(24) groups, respectively. Sand holly (*Ammopiptanthus mongolicus*) showed a tendency of increasing and then decreasing after either 12 h or 24 h of IAA immersion, in which the maximum root length was 8.35 in the IAA-immersed T2(12) group, which increased by 25.38% and 25.94% compared with the CK(12) and CK(24) groups, respectively.

The root length of all fractions of (*Sarcozygium xanthoxylon*) after 12 h of IAA immersion showed a trend of first increasing decreasing, and then increasing, in which the T1 group had the maximum root length of 13.32 cm, which was 78.55% and 93.89% higher than the CK(12) and CK(24) groups, respectively, and all of them were significantly greater than that of all fractions immersed for 24 h (*p* < 0.05). White thorn (*Nitraria tangutorum*) showed a trend of decreasing and then increasing in all fractions after 12 h of IAA soaking, with the maximum root length of 13.54 cm in T3 group, which increased by 87.02% and 76.53% compared to CK(12) and CK(24) groups, respectively. Sand holly (*Ammopiptanthus mongolicus*) showed a tendency of increasing and then decreasing after either 12 h or 24 h of IAA immersion, in which the maximum root length was 8.35 in the IAA-immersed T2(12) group, which increased by 25.38% and 25.94% compared with the CK(12) and CK(24) groups, respectively.

The leaf length of all components of *Sarcozygium xanthoxylon* and *Ammopiptanthus mongolicus* after IAA immersion for 12 h and 24 h showed a trend of first increasing and then decreasing, while the leaf length of *Sarcozygium xanthoxylon* showed a gradual upward trend. The root length of the T1 group was 13.32 cm, which was 78.55% and 93.89% higher than that of the CK (12) and CK (24) groups, respectively, and was significantly higher than that of all components soaked for 24 h (*p* < 0.05). After 12 h of IAA immersion, all components of *Nitraria tangutorum* decreased first and then increased, and the root length of the T3 group was 13.54 cm, which was 87.02% and 76.53% higher than that of the CK (12) and CK (24) groups, respectively. *Ammopiptanthus mongolicus* increased first and then decreased after IAA immersion for 12 h or 24 h, and the root length of the IAA-soaked T2 (12) group was 8.35, which was 25.38% and 25.94% higher than that of the CK (12) and CK (24) groups, respectively. The maximum leaf length of the T1 (12) group was 4.4 cm, which was 54.93% and 29.79% higher than that of the CK (12) and CK (24) groups, respectively, and there was no significant difference between CK (24), T1 (24), T2 (24), and T3 (24) of *Sarcozygium xanthoxylon* (*p* > 0.05). The maximum leaf length component of *Nitraria tangutorum* was T3 (12), which was 31.78% and 54.10% higher than that of CK (12) and CK (24), respectively. The highest value of T1 (24) of *Ammopiptanthus mongolicus* was 2.48 cm, which was 47.62% and 22.97% higher than that of the CK (12) and CK (24) groups, respectively.

### Effects of IAA immersion and exogenous melatonin treatment on root viability of three desert plants at different times

**Figure 3.**
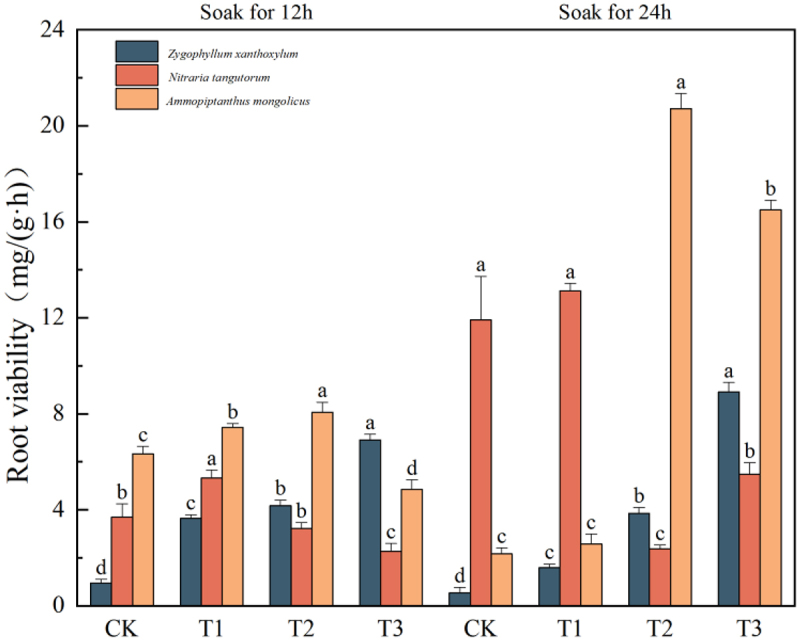
Trends in root vigor of three desert plant species in response to different times of IAA immersion and exogenous melatonin treatment.

It can be seen from Figure 3 that the highest value of root activity of the three desert plants was in the component of IAA immersion for 24 h, and the highest peak root activity of *Sarcozygium xanthoxylon* was 8.91 mg/(g·h) in the T3 (24) group, which showed a gradual upward trend with the increase of melatonin concentration. The highest peak root activity of *Nitraria tangutorum* was 13.13 mg/(g·h) in the T1 (24) group, which showed a trend of increasing and then decreasing and then increasing with the increase of melatonin concentration. The highest peak root activity of *Ammopiptanthus mongolicus* was 20.71 mg/(g·h) in the T2 (24) group, which first increased and then decreased with the increase of melatonin concentration. The results showed that high concentrations of melatonin (MT) could inhibit the root viability of plants, and the effect of IAA soaking for 24 h was the most obvious.

### Effect of IAA immersion and exogenous melatonin treatment at different times on fresh and dry weights of three desert plants

**Figure 4.**
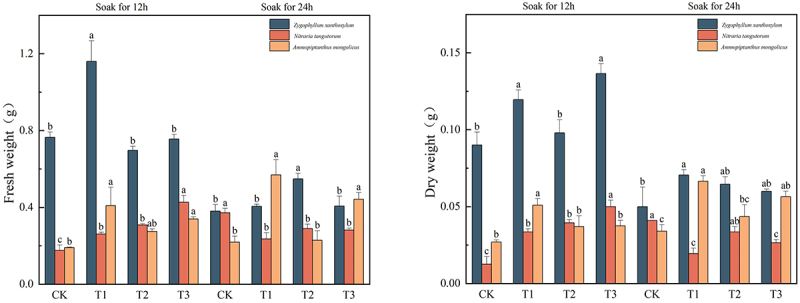
Effect of IAA immersion and exogenous melatonin treatment at different times on fresh and dry weight of three desert plants.

In Figure 4, the fresh weight of *Sarcozygium xanthoxylon* was T1 (12), and the highest value was 1.16 g, which was 51.63% and 205.26% higher than that of the CK (12) and CK (24) groups, respectively. The fresh weight of *Nitraria tangutorum* was 0.43 g under the T3 (12) solution, which was 138.89% and 2% higher than that of the CK (12) and CK (24) groups, respectively, and the fresh weight increased with the increase of melatonin concentration among the groups after IAA immersion for 12 h. After 24 h of IAA immersion, the group showed a trend of decreasing and increasing. The maximum fresh weight of *Ammopiptanthus mongolicus* was 0.57 g at T1 (24), and the maximum fresh weight of *Ammopiptanthus mongolicus* increased by 200% and 159.09% compared with the CK (12) and CK (24) groups, respectively.

The maximum dry weight of Sarcozygium xanthoxylon was 0.137 g after T1 (12), and there was no significant difference in the dry weight of *Sarcozygium xanthoxylon* (*p*> .05). The trend of all components of dry weight of *Sarcozygium xanthoxylon* was the same as that of fresh weight. The maximum dry weight of *Nitraria tangutorum* was divided into T3 (12) group, with a maximum value of 0.05 g, which was 284.62% and 21.95% higher than that of CK (12) and CK (24) groups, respectively. The maximum dry weight of *Ammopiptanthus mongolicus* was divided into the T1 (24) group, and its maximum value was 0.067 g, which was 148.15% and 97.06% higher than that of the CK (12) and CK (24) groups, respectively. Whether the IAA was soaked for 12 h or 24 h, the trend was to increase first and then decrease.

### Effects of IAA immersion and exogenous melatonin treatment at different times on chlorophyll a, chlorophyll b, carotenoids, and total chlorophyll in three desert plants

**Figure 5.**
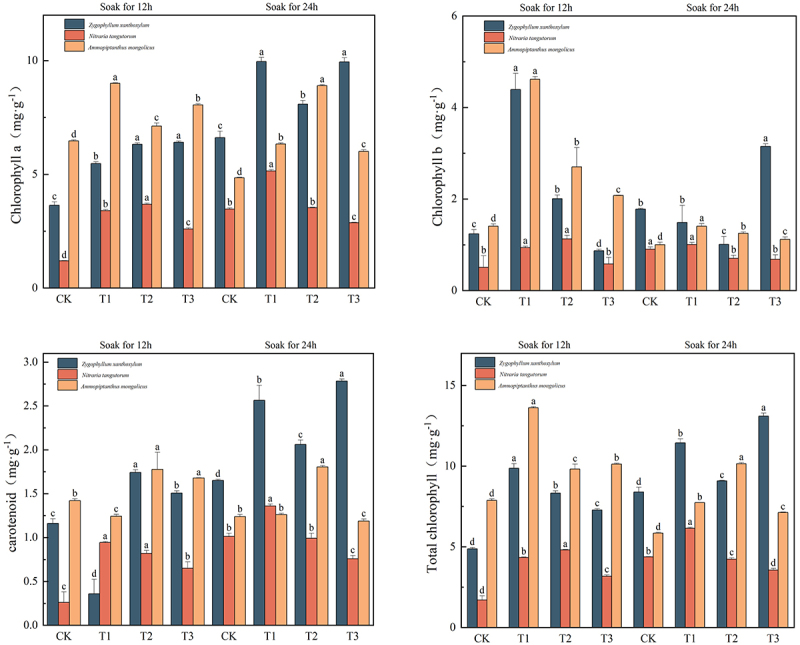
Changes in chlorophyll a, chlorophyll b, carotenoids, and total chlorophyll content of three desert plants at different times of IAA immersion and exogenous melatonin treatment.

As can be seen in Figure 5, the maximum chlorophyll content of *Sarcozygium xanthoxylon*, white thorn, and sand holly was 9.96 mg·g^−1^, 5.15 mg·g^−1^, and 9.00 mg·g^−1^, respectively. The maximum chlorophyll component of *Sarcozygium xanthoxylon* was 173.63% and 50.68% higher than that in the T1 (24) group compared with the CK (12) and CK (24) groups, respectively. The maximum chlorophyll of *Nitraria tangutorum* was divided into T1 (24) group, which increased by 329.17% and 48.14% compared with CK (12) and CK (24) groups, respectively. The maximum green component of *Ammopiptanthus mongolicus* was in the T1 (12) group, which increased by 39.10% and 85.57%, respectively, compared with the two CKs.

The maximum chlorophyll b content of *Sarcozygium xanthoxylon*, *Nitraria tangutorum*, and *S. chinensis* was 4.39 mg·g^−1^, 1.134.39 mg·g^−1^ and 4.62 mg·g^−1^, respectively. Compared with the CK (12) and CK (24) groups, the maximum chlorophyll b of *Sarcozygium xanthoxylon* increased by 254.03% and 146.63%, respectively. Compared with the CK (12) and CK (24) groups, the maximum chlorophyll b component o*f Nitraria tangutorum* increased by 127.57% and 25.56%, respectively, regardless of whether the IAA was immersed in 12 h or 24 h, the changing trend in the group was first increased and then decreased. Compared with the CK (12) and CK (24) groups, the maximum chlorophyll b component T1 (24) of *Ammopiptanthus mongolicus* significantly increased by 228.57% and 360% (*p* < 0.05), respectively, and were more significant than all components of IAA immersion for 24 h.

The maximum carotenoid content of *Sarcozygium xanthoxylon*, *Nitraria tangutorum*, and *S. chinensis* was 2.56 mg·g^−1^, 1.36 mg·g^−1,^ and 1.81 mg·g^−1^. The maximum carotenoid content of *Sarcozygium xanthoxylon* was divided into T1 (24) group, which increased by 120.69% and 55.15% compared with CK (12) and CK (24) groups, respectively, and the carotenoid content of T1 (24), T2 (24) and T3 (24) groups was greater than that of all components soaked in IAA for 12 h. Compared with the CK (12) and CK (24) groups, the maximum carotenoid content of *Nitraria tangutorum* was divided into T1 (24) group, which increased by 423.08% and 36%, respectively. The maximum carotenoid fraction of *Ammopiptanthus mongolicus* was divided into T2 (24) group, which increased by 26.76% and 45.16% compared with CK (12) and CK (24) groups and increased by 42.86% and 51.26% compared with T1 (24) and T3 (24), respectively.

The maximum value of the total chlorophyll in *Sarcozygium xanthoxylon* was 13.10 mg·g^−1^ in the T3 (24) group. Compared with the CK (12) and CK (24) groups, the increase was 168.44% and 56.14%, respectively, and the CK (24) group increased by 71.39% compared with the CK (12) group. The maximum value of total chlorophyll in *Nitraria tangutorum* was in the T1 (24) group, and the maximum value was 6.15 mg·g^−1^. Compared with the CK (12) and CK (24) groups, it increased by 261.76% and 40.41%, respectively, and compared with the T2 (24) and T3 (24) groups, it increased by 45.39% and 72.75%, respectively. The maximum value of total chlorophyll in *Ammopiptanthus mongolicus* was in the T1(12) group, with a maximum value of 13.61 mg·g^−1^. Compared with the CK (12) and CK (24) groups, which increased by 72.72% and 132.65%, respectively, the same treatment of IAA immersion for 12 h increased by 38.59% and 34.35% compared with T2 (12) and T3 (12), respectively.

### Effects of IAA immersion and exogenous melatonin treatment at different times on osmoregulation in three desert plants

**Figure 6.**
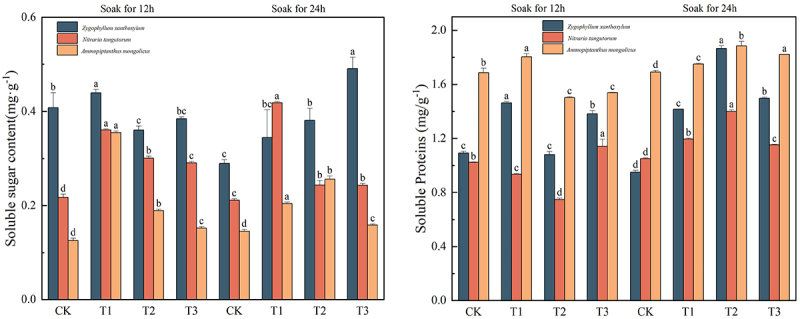
Changes in the content of soluble sugars and soluble proteins in three desert plants by different times of IAA immersion and exogenous melatonin treatment.

As can be seen in Figure 6 the highest content of soluble sugar and soluble protein in Sarcozygium xanthoxylon was T3 (24) group (0.491 mg·g-1) and T2 (24) group (1.866 mg·g-1), respectively. Compared with the CK (12) and CK (24) groups, the highest soluble sugar content of *Sarcozygium xanthoxylon was* increased by 20.34% and 69.31%, respectively. Compared with the CK (12) and CK (24) groups, the soluble protein content of *Sarcozygium xanthoxylon* increased by 71.56% and 97.05%, respectively. The components with the highest content of soluble sugar and soluble protein in *Nitraria tangutorum* were the T1 (24) group (0.418 mg·g^−1^) and the T2 (24) group (1.399 mg·g^−1^), respectively. Compared with the two CK groups, the highest soluble sugar content of *Nitraria tangutorum* increased by 92.63% and 98.10%, respectively, and the changes of soluble sugar in the group increased first and then decreased. Compared with the CK (12) and CK (24) groups, the highest soluble protein content of *Nitraria tangutorum* was increased by 36.62% and 333.11%, respectively. *Ammopiptanthus mongolicus* had the highest content of soluble sugar and soluble protein, respectively, in the T1 (12) group (0.355 mg·g^−1^) and T2 (24) group (1.855 mg·g^−1^). Compared with the CK (12) and CK (24) groups, the highest soluble sugar content of *Ammopiptanthus mongolicus* was increased by 181.75% and 143.15%, respectively. Compared with the CK (12) and CK (24) groups, the most significant soluble protein content of *Ammopiptanthus mongolicus* increased by 11.74% and 11.41%, respectively, and the changes of soluble sugar and soluble protein in the group showed a trend of first increasing and then decreasing.

**Figure 7.**
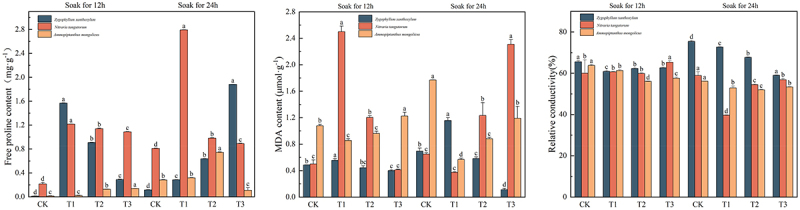
Changes in the content of free proline, malondialdehyde, and relative electrical conductivity of three desert plants at different times of IAA immersion and exogenous melatonin treatment.

In Figure 7, the maximum proline content of *Sarcozygium xanthoxylon* was 1.880 mg·g^−1^, and the component was divided into the T3 (24) group. Compared with the CK (12) and CK (24) groups, which increased by 1392.06% and 1520.69%, respectively, the value change of IAA soaked for 12 h showed a trend of first increasing and then decreasing, while the value change of IAA soaked for 24 h showed a gradual increase trend. The maximum proline content of *Nitraria tangutorum* was 2.793 mg·g^−1^, and the group was divided into the T1 (24) group. Compared with the CK (12) and CK (24) groups, it increased by 1205.14% and 171.08%, respectively. The maximum proline content of *Ammopiptanthus mongolicus* was 2.793 mg·g^−1^, and the component was divided into the T1 (24) group. Compared with the CK (12) and CK (24) groups, the increase was 447.79% and 165.12%, respectively.

The higher the malondialdehyde values and relative conductivity, the worse the plant’s stress resistance. The minimum values of malondialdehyde and relative conductivity of *Sarcozygium xanthoxylon* were in the T3 (24) group, and their values were 0.122 μmol·g^−1^ and 0.590%, respectively. Compared with the two CK groups, the values of malondialdehyde in the T3(24) group decreased by 77% and 83.86%, respectively, and the changes in the group showed a trend of first increasing and then decreasing. The lowest group of the relative conductivity phase of *Sarcozygium xanthoxylon* was 9.92% and 21.75% lower than that of the two CK groups, respectively. The minimum values of malondialdehyde and relative conductivity of *Nitraria tangutorum* were 0.373 μmol·g^−1^ and 0.396% in the T1 group immersed in IAA for 24 h. The malondialdehyde in the T1 group immersed in IAA for 24 h decreased by 25.55% and 43.53%, respectively, and the lowest group of the relative conductivity phase of *Nitraria tangutorum* decreased by 33.89% and 32.77%, respectively, compared with the two CK groups. The minimum values of malondialdehyde and relative conductivity of *Ammopiptanthus mongolicus* were in the T1 (24) and T2 (24) groups, respectively, and their values were 0.568 μmol·g-1 and 0.520%, respectively. The minimum malondialdehyde values were 90.14% and 67.89% lower than those of CK (12) and CK (24). The lowest group of relative conductivity phase of *Ammopiptanthus mongolicus* was 18.50% and 7.31% lower than that of CK (12) and CK (24), respectively.

### Effects of IAA immersion and exogenous melatonin treatment at different times on antioxidant activity of three desert plants

The highest content of peroxidase, catalase, and superoxide dismutase in *Sarcozygium xanthoxylon* was in the T3 (24) group. The maximum content of peroxidase in *Sarcozygium xanthoxylon* was 131.67 U·g-1·min-1, which were CK (12) and CK (24), respectively, 10.82 times and 1.97 times of the group, and all components of IAA soaked for 24 h were greater than those of IAA soaked for 12 h. The maximum catalase content of *Sarcozygium xanthoxylon* was 13.26 U·g-1·min-1, 4.69, and 4.03 times higher than that of CK (12) and CK (24) groups, respectively. The maximum superoxide dismutase content of *Sarcozygium xanthoxylon* was 55.28 U·g-1·min-1, which was 1.45 and 1.37 times higher than that of the two CK groups, respectively.

The maximum peroxidase and superoxide dismutase values in *Nitraria tangutorum* were in the T1 (24) group. In contrast, the maximum value of catalase in the T3 (24) group and the maximum value of peroxidase in *Nitraria tangutorum* was 943.33 U·g-1·min-1, which was 40.06 and 51.46 times higher than that of the CK (12) and CK (24) groups, respectively, and was significantly higher than that of T2 (24) and T3 (24) increased by 140.85% and 301.42%, respectively. The maximum superoxide dismutase content of *Nitraria tangutorum* was 63.57 U·g-1·min-1, which was 1.49 and 1.3 times higher than that of the CK (12) and CK (24) groups, respectively. The maximum catalase content of *Nitraria tangutorum* was 55.53 U·g-1·min-1, which was 9.61 and 3.18 times higher than that of the CK (12) and CK (24) groups, respectively.

The maximum values of peroxidase and catalase in *Ammopiptanthus mongolicus* were both in the T1 (24) group, and the maximum peroxidase content in *Ammopiptanthus mongolicus* was 403.33 U·g-1·min-1, which was 1.85 and 8.35 times higher than that in the CK (12) and CK (24) groups, respectively. The maximum value of catalase in *Ammopiptanthus mongolicus* was 102 U·g-1·min-1, which was 5.77 and 3.13 times higher than that of the CK (12) and CK (24) groups, respectively.

**Figure 8.**
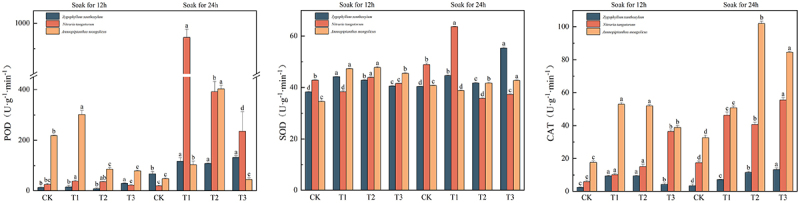
Variation in peroxidase, catalase, and superoxide dismutase content in three desert plants.

### Pearson correlation coefficient between the indexes of three desert plants

**Figure 9.**
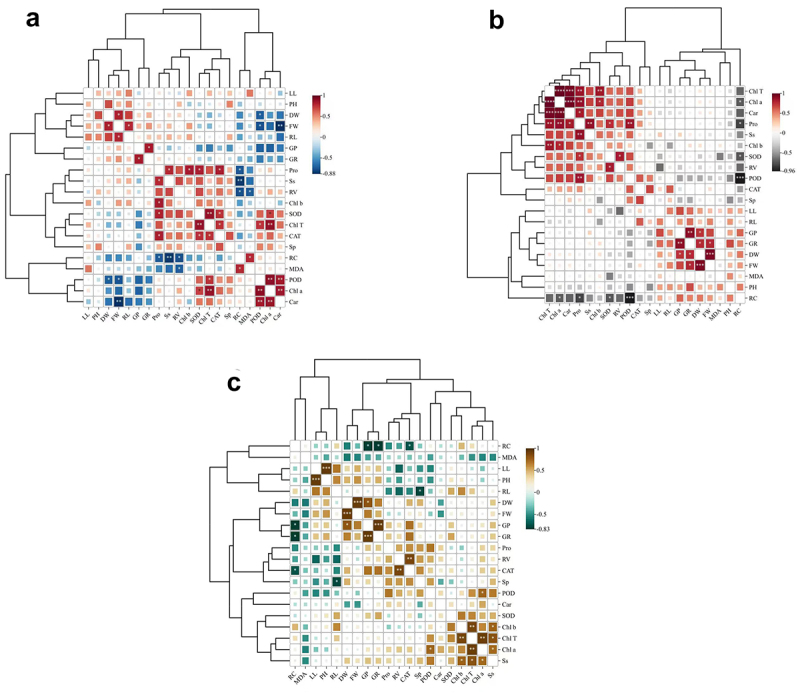
Shows the cluster analysis of the correlation of the indicators of three desert plants: a is *sarcozygium xanthoxylon*, B is *nitraria tangutorum*, and C is *Ammopiptanthus mongolicus*.

**Figure 10.**
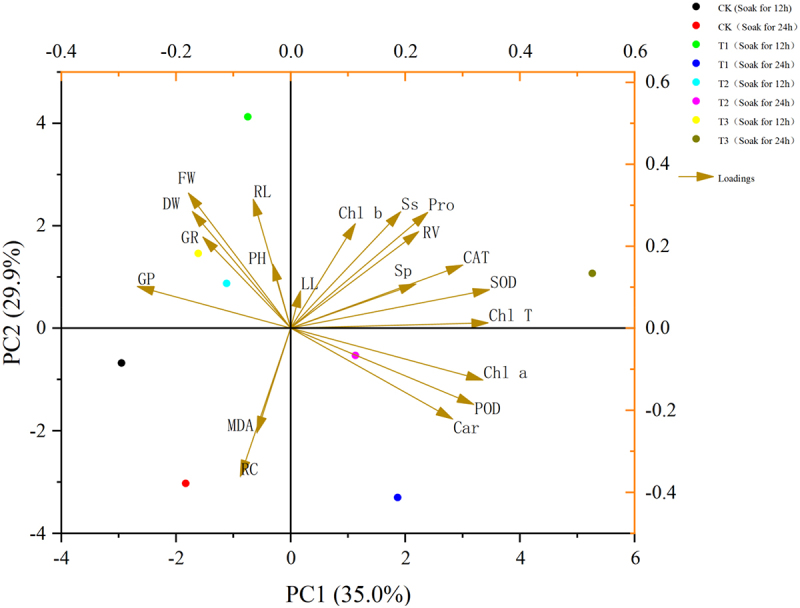
Shows the *sarcozygium xanthoxylon* PCA analysis.

The results of correlation analysis of the indicators of *Sarcozygium xanthoxylon*, *Nitraria tangutorum*, and *Ammopiptanthus mongolicus* (Figures 8, 9 and Figure 10) showed that there was a very significant positive correlation between the indicators *Sarcozygium xanthoxylon* was positively correlated with Chl T (*p* < 0.01) with a correlation coefficient of 0.88, while Chl a was positively correlated with Chl T and POD with correlation coefficients of 0.88 and 0.90, respectively. The correlation coefficients between Chl a and Car and Chl T were 0.86 and 0.88, respectively. RC was negatively correlated with Ss with a negative correlation coefficient of −0.85, and Car was negatively correlated with FW with a negative correlation coefficient of −0.88. Among them, Chl a and Chl T, Car, and Chl T and Car were significantly positively correlated with 0.99, 0.97, and 0.95, respectively, while POD was significantly negatively correlated with RC with a negative correlation coefficient of −0.96. Among them, the GP of *Ammopiptanthus mongolicus* was significantly positively correlated with GR, LL, and PH, and DW was significantly positively correlated with FW, with positive correlation coefficients of 0.94, 0.94, and 0.93, respectively. In contrast, RC was negatively correlated with GP, GR, and CAT, with negative correlation coefficients of −0.83, −0.82, and −0.73, respectively.

### Principal component analysis

A comprehensive analysis of the 20 indicators of overlord (*Sarcozygium xanthoxylon*) was carried out by principal component analysis to evaluate the overall effect of different indicators on overlord (*Sarcozygium xanthoxylon*), and the results showed (Table 3) that the rotational loading of 6.413, the maximum contribution rate of 32.063% after rotation as well as the first four principal components had a cumulative contribution of 88.226%, which exceeded 85%. The first three components can be extracted as the indicators for the next step of principal component analysis.

**Table 3.** Shows the component matrix scores of *sarcozygium xanthoxylon.*

Index	PC1	PC2	PC3	PC4
GR	−0.118	0.063	−0.044	0.008
GP	−0.148	0.032	−0.119	−0.041
PH	0.052	−0.04	0.387	−0.084
LL	−0.044	0.01	0.159	0.14
RL	0.034	−0.101	0.064	0.361
RV	0.03	0.153	0.131	−0.166
FW	−0.109	0.021	0.067	0.137
DW	−0.067	0.01	0.219	−0.018
Chl a	0.152	0.002	0.042	0.027
Chl b	−0.065	0.093	−0.218	0.291
Car	0.139	0.019	0.028	−0.155
Chl T	0.101	0.047	−0.068	0.163
Ss	−0.01	0.154	0.026	−0.035
Sp	0.112	0.007	0.246	0.016
Pro	−0.017	0.154	−0.105	0.118
MDA	0.079	−0.194	0.035	0.22
RC	0.055	−0.166	−0.07	0.092
POD	0.151	−0.005	0.009	0
CAT	0.06	0.103	0.001	0.064
SOD	0.059	0.131	−0.1	0.035
Rotational loads	6.413	5.762	2.811	2.66
Post-rotation contribution rate	32.063	28.81	14.053	13.3
Post-rotation cumulative contribution rate	32.063	60.873	74.926	88.226

Based on the four principal components analyzed by SPSS, the final scores of *Sarcozygium xanthoxylon* under different treatments were calculated to select the optimal treatments to improve its salt tolerance.

Y = 32.063/88.226*FAC1_1+28.810/88.226*FAC2_1+14.053/88.226*FAC3_1+13.300/88.226 * FAC4_1.

**Table 4.** Shows the final scores of *sarcozygium xanthoxylon* in each group under different treatments.

Serial number	Group	Score
①	CK(12)	−0.7
②	T1(12)	0.19
③	T2(12)	−0.26
④	T3(12)	−0.05
⑤	CK(24)	−0.69
⑥	T1(24)	0.34
⑦	T2(24)	0.31
⑧	T3(24)	0.85

In Table 4,*Sarcozygium xanthoxylon* grew and developed best in saline soil under treatment using 100 μmol/L IAA soaked for 24 h and a melatonin (MT) concentration of 300 μmol/L.

**Figure 11.**
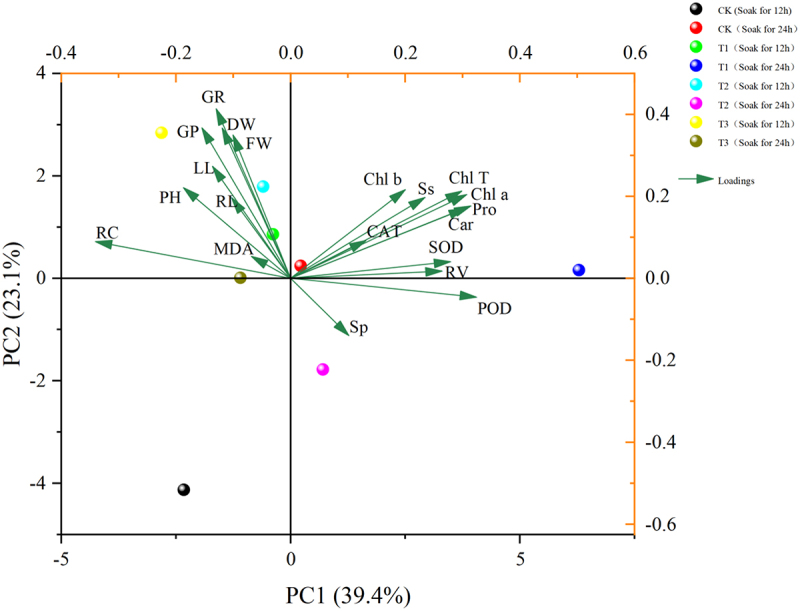
Shows the PCA analysis of *nitraria tangutorum*.

As can be seen in Figure 11, the20 indicators of *Nitraria tangutorum*were comprehensively analyzed using principal component analysis to evaluate the overall effect of different indicators on a *Nitraria tangutorum*, and the results showed (Table 5) that the rotational loading of 6.965, the maximum contribution of 34.825% after rotation, and the cumulative contribution of the first four principal components were 85.123%, which is more than 85%. The first four components can be extracted as various indicators for the next step of principal component analysis.

**Table 5.** Shows the component matrix scores of the indicators of *nitraria tangutorum.*

Index	PC1	PC2	PC3	PC4
GR	0.031	0.213	−0.025	−0.037
GP	0.017	0.192	−0.031	−0.022
PH	0.019	0.065	−0.041	0.184
LL	−0.031	0.127	0.263	−0.005
RL	0.043	0.064	0.11	0.266
RV	0.059	0.028	−0.058	−0.214
FW	−0.008	0.232	0.064	−0.172
DW	0.016	0.206	−0.008	−0.078
Chl a	0.149	0.024	−0.005	0.036
Chl b	0.149	0.018	−0.213	0.075
Car	0.135	0.02	0.013	0.008
Chl T	0.154	0.024	−0.04	0.044
Ss	0.144	−0.001	−0.02	0.137
Sp	−0.041	−0.034	0.3	−0.062
Pro	0.135	0.021	0.057	0.019
MDA	0.076	−0.116	−0.023	0.452
RC	−0.086	0.089	−0.075	0.008
POD	0.084	−0.069	0.151	0.015
CAT	0.044	0.006	0.317	0.114
SOD	0.058	0.05	−0.044	−0.256
Rotational loads	6.965	4.809	2.819	2.432
Post-rotation contribution rate	34.825	24.045	14.094	12.16
Post-rotation cumulative contribution rate	34.825	58.87	72.964	85.123

Based on the four principal components analyzed by SPSS, the final scores of *Nitraria tangutorum* under different treatments were calculated to select the optimal treatment to improve its salt tolerance.

Y=34.825/85.123∗FAC1_1+24.045/85.123∗FAC2_1+14.094/85.123∗FAC3_1+12.160/85.123∗FAC4_1

**Table 6.** Shows the final scores of nitraria tangutorum in each group under different treatments.

Serial number	Group	Score
①	CK(12)	−1.17
②	T1(12)	0.15
③	T2(12)	0.21
④	T3(12)	0.42
⑤	CK(24)	−0.23
⑥	T1(24)	0.57
⑦	T2(24)	−0.16
⑧	T3(24)	0.22

In Table 6,*Nitraria tangutorum* developed best in saline soil under treatments using 100 μmol/L IAA immersion for 24 h and a melatonin (MT) concentration of 100 μmol/L.

**Figure 12.**
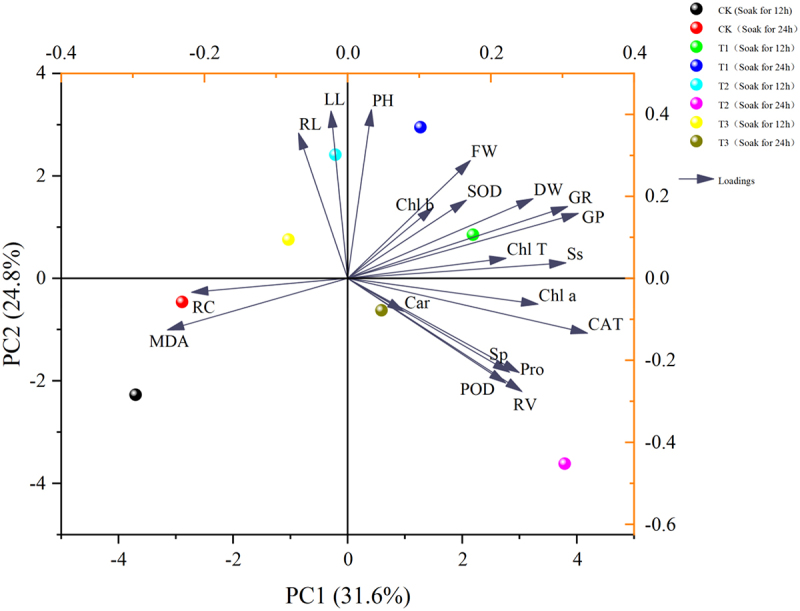
Shows the PCA analysis of ammopiptanthus mongolicus.

As can be seen in Figure 12, a comprehensive analysis of 20 indicators of *Ammopiptanthus mongolicus* was conducted using principal component analysis to evaluate the overall effect of different indicators on *Ammopiptanthus mongolicus*, and the results showed (Table 7) that the rotational loading of 5.035, the maximum contribution of 25.175% after rotation, and the cumulative contribution of the first four principal components of 88.474% exceeded 85%. The first four components can be extracted and used as indicators for the next step of principal component analysis.

**Table 7.** Shows the component matrix scores for the indicators of *ammopiptanthus mongolicus.*

Index	PC1	PC2	PC3	PC4
GR	−0.051	0.212	0	−0.083
GP	−0.022	0.181	0.004	0.02
PH	−0.165	0.088	0.001	0.043
LL	−0.183	−0.008	0.098	−0.091
RL	−0.177	0.087	−0.019	0.009
RV	0.135	0.087	0	−0.084
FW	−0.053	0.043	0.031	0.249
DW	−0.003	0.078	0.019	0.238
Chl a	0.043	0.001	0.176	−0.077
Chl b	−0.053	−0.086	0.207	0.057
Car	−0.027	0.119	0.019	−0.365
Chl T	−0.001	−0.043	0.211	−0.018
Ss	0.041	−0.02	0.181	0.109
Sp	0.174	−0.023	0.017	0.232
Pro	0.116	0.101	−0.011	−0.081
MDA	0.003	−0.029	−0.111	−0.106
RC	0	−0.228	0.111	0.087
POD	0.139	−0.051	0.128	0.012
CAT	0.093	0.152	−0.003	−0.048
SOD	−0.093	0.084	0.105	−0.151
Rotational loads	5.035	5.032	4.883	2.745
Post-rotation contribution rate	25.175	25.161	24.416	13.723
Post-rotation cumulative contribution rate	25.175	50.336	74.752	88.474

Based on the four principal components analyzed by SPSS, the final scores of *Ammopiptanthus mongolicus* under different treatments were calculated to select the optimal treatments to improve its salt tolerance.

Y=25.175/88.474∗FAC1_1+25.161/88.474∗FAC2_1+24.416/88.474∗FAC3_1+13.723/88.474∗FAC4_1

**Table 8.** Shows the final scores of *ammopiptanthus mongolicus* in each group under different treatments.

Serial number	Group	Score
①	CK(12)	−0.53
②	T1(12)	0.53
③	T2(12)	−0.33
④	T3(12)	−0.34
⑤	CK(24)	−0.55
⑥	T1(24)	0.21
⑦	T2(24)	0.81
⑧	T3(24)	0.21

In Table 8,*Ammopiptanthus mongolicus* grew and developed best in saline soils under treatments using 100 μmol/L IAA soaked for 24 h and melatonin (MT) at 200 μmol/L.

## Comprehensive evaluation of the affiliation function

**Table 9.** Shows the final scores of three desert plants in each group under different treatments.

	Subordinative function value					
Desert plants	X1	X2	μ1	μ2	D	Ranking
*Sarcozygium xanthoxylon*	1.1384	−0.1932	1	0.3530	0.7833	1
*Nitraria tangutorum*	−0.7365	−0.8893	0	0	0	3
*Ammopiptanthus mongolicus*	−0.4019	1.0825	0.1785	1	0.4537	2
weight	w1 = 0.665	w1 = 0.335				

In Table 9, In descending order, the salinity tolerance of the three desert plants is *Sarcozygium xanthoxylon* > *Ammopiptanthus mongolicus* > *Nitraria tangutorum*, respectively, as evaluated by affiliation function.

## Disscussion

**Figure 13.**
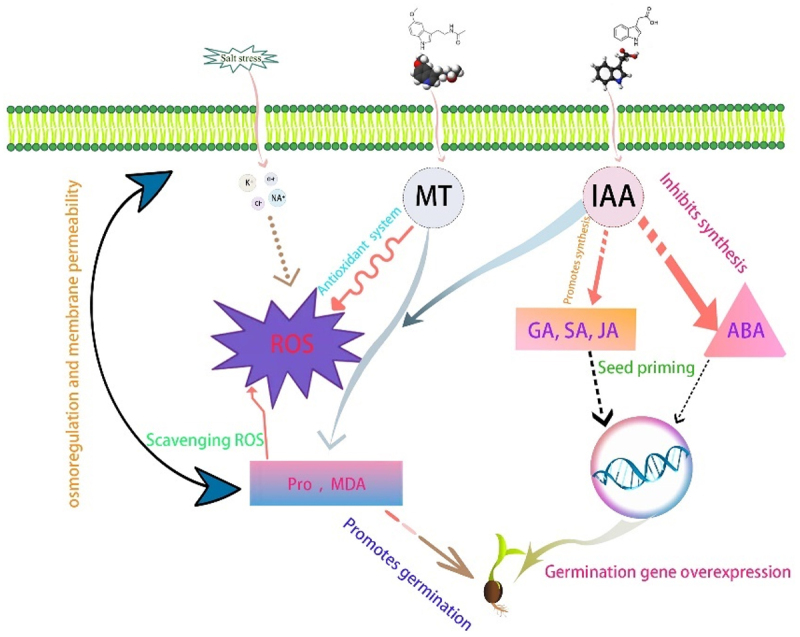
Illustrates the mechanism of IAA and MT inside the seed.

Salt stress is one of the abiotic stresses affecting plant growth, inhibiting the growth and development of plant tissues and organs and seed germination, and the application of plant growth substances or exogenous chemicals can effectively reduce the damage of salt stress to plants and enhance the salt resistance of plants.^34^ Seed germination is an initial phenotypic feature of the plant life cycle and is associated with biological adaptation.^35^ In Figure 13, we explored the mechanism of seed initiation by two hormones. This study found that the germination rate and germination potential of the three desert plants were relatively low when they were grown in the same saline-alkali soil without treatment. However, the germination rate and germination potential of exogenous melatonin were higher when soaked in IAA and applied, indicating that exogenous IAA and melatonin could alleviate the damage caused by salinity to seed germination, and high concentration of melatonin showed an inhibitory effect on seeds. In contrast, low concentrations of melatonin showed a promoting effect. Similar observations have been reported in other plants, where soaking buckwheat seeds with MT can attenuate the inhibitory effect of high temperature on seed germination, and seeds soaked with 200 μM MT germinate faster than seeds not treated with MT under the same high-temperature stress.^36^ José Luis Castañares et al.^37^ found how exogenous melatonin induces biochemical changes in melon plants, thereby increasing salt stress tolerance. Concentrations ranged from 10 to 50 μmol· L^−1^ appeared to be optimal for increased germination under salt stress. When considering seedling growth, 50 μmol· L^−1^ melatonin is applied by seed absorption and watering. Tianlun Zhao et al.^3^ found that 20 mg L^−1^IAA significantly improved seed germination, promoted seedling growth, and regulated the endogenous plant hormone content of germinated seeds and seedlings by regulating the following biological processes. The results indicated that IAA soaking could promote the germination rate of seeds. Zhao Yongteng et al.^38^ found that MT (10–200 uM) or GABA (10–200 uM) alone significantly mitigated the effects of Cd stress on tomato seedlings, which showed a significant increase in GR, GP, GI, and VI compared to Cd-only treated seedlings. TYAGI SHWETA et al.^9^ found that treatment with growth hormones (1×10-8 M IAA, 1 × 10-5 M of Kn and 1 × 10-4 M of GA) was found to promote seed germination and seedling growth in chickpea. Guangwu Zhao et al.^39^ found that IAA and GA3 co-treatments were used to enhance the germination rate of cedar (*Cunninghamia lanceolata*) seeds, and that IAA and GA3 had the most significant promotional effects at concentrations of 10–4 M and 10–5 M, respectively. Li Ruiqing et al.^40^ found that gibberellin (GA) synthesis was restored to or even exceeded the CK level in MT pre-soaking treatments, whereas abscisic acid (ABA) content decreased compared to Cu-stressed seeds, suggesting crosstalk between MT and other phytohormones such as GA and ABA. Wiem Mnafgui et al.^41^ found that application of IAA triggered *Trigonella Foneum graecum* L. in the presence of lead excess, increased catalase, glutathione (GSH), ascorbate peroxidase (APX), flavonoids, and phenolics in comparison to other treatments, and that IAA has a greater efficiency, which reduces reactive oxygen species (ROS) activity and increases specific phenolics by up-regulating the mechanism of antioxidant system. Liexiang Huangfu et al.^42^ found that the antioxidant capacity of rice seeds under salt stress was enhanced by the application of melatonin pretreatment, with increased levels of indole-3-acetic acid (IAA) and decreased levels of abscisic acid (ABA). The enhanced antioxidant activity was also supported by metabolomic assays, i.e., melatonin pretreatment increased the levels of non-enzymatic antioxidant organic acids and amino acids. Li Junpeng et al.^43^ found that seeds pretreated with melatonin contained high levels of melatonin and gibberellin (GA), low levels of abscisic acid (ABA), and high levels of amylase and α-amylase activities during seed germination, and melatonin treatment up-regulated the expression of key genes for GA biosynthesis (GA20ox and GA3ox) and down-regulated key genes for ABA biosynthesis (Lb NCED1 and Lb NCED3) expression, leading to changes in GA and ABA levels during seed germination. Chen Li et al.^44^ found that pretreatment of cotton seeds with 20 μM exogenous melatonin under salt stress increased cotton germination rate and hypocotyl length as well as endogenous melatonin content during seed germination. Melatonin regulates the expression of ABA and GA genes in plant signal transduction pathways, induces radicle development and seed germination, and alleviates dormancy. Zhicheng Hu et al.^45^ found that melatonin pretreatment altered the expression of genes involved in redox and cell wall formation processes, and melatonin also increased the level of glutathione (GSH), which chelates excess CU^2+^ and inhibits jasmonic acid biosynthesis to promote the development of melon roots under Cu stress.

The accumulation of biomass is an essential manifestation of adaptation to stress, and biomass is one of the crucial indicators of plant salt tolerance.^46^ After soaking and applying exogenous melatonin using IAA, the plant height, root length, leaf length, dry weight, and fresh weight of the three desert plants were increased. The results of field experiments by Zhiqin Chen et al.^47^ showed that IAA alone could effectively increase the biomass and photosynthetic pigment of S. alfredii. Hu Diandian et al.^48^ found that caryophyllus sprayed at an appropriate concentration of MT (100 μM) could reduce plant damage by increasing biomass, reducing oxidative damage, coordinating osmotic accumulation, and activating the antioxidant defense system, which was consistent with the results obtained in this study of overlord biomass.

Roots are vital organs for absorbing and transporting water and mineral nutrients.^49^ Mansfield et al.^50^ found that indole-3-acetic acid (IAA), which is considered the primary auxin in plants, stimulates growth through cell elongation and lateral root formation, which may support greater uptake of minerals, and it also acts as a signaling molecule under stress. Root vigor is an important physiological indicator of plant growth robustness and stress resistance,^51^ and this study showed that using IAA soaking and applying exogenous melatonin could significantly improve the root vigor of three desert plants. Zhaoyang Li et al.^52^ found that treatment significantly improved root viability in Thalassia Hemprichii using the IAA component compared to the CK group. These results indicated that IAA played a promoting role in improving root viability. The results of this study showed that after 24 h of IAA immersion, *Sarcozygium xanthoxylon* had the highest root activity when applying 300 μmol/L melatonin, 100 μmol/L melatonin was the highest in white thorn, and 100 μmol/L melatonin was the highest in *S. saxon*. A similar study has also reported that Chu Yutan et al.^53^ found that exogenous MT addition could significantly increase rice root activity, while Sb stress significantly decreased rice seedling root activity. After Sb stress, the root activity of rice decreased by 48.7% compared with CK, while the exogenous addition of MT increased the root activity of rice seedlings under Sb stress by 44.1%, which was consistent with the results of this study.

Chlorophyll a and b are important photosynthetic pigments that represent the growth state of plants, reflect environmental or biological stresses, and are essential parameters for monitoring plant health.^54^ This study showed that the chlorophyll content of three desert plants could be significantly increased by soaking with IAA and applying exogenous melatonin. Similar studies have also demonstrated that the application of exogenous MT increases chlorophyll content, and Menhas et al.^55^ found that MT can promote the growth and quality of Brassica napus seedlings under cadmium toxicity conditions and improve photosynthetic pigment content and cadmium uptake by leaf tissues. In the results of Jawaria Jameel et al.^56^ chlorophyll a, b, and total chlorophyll were significantly reduced at different levels of salt stress in all eggplant varieties. Therefore, similar to this experiment, saline-alkali soil reduced the chlorophyll content of three desert plants.

The relative conductivity and MDA levels reflect the degree of toxicity and damage to plant biofilms caused by salt stress. After IAA immersion and exogenous melatonin treatment, the MDA and relative conductivity of the three desert plants were significantly reduced. Recent studies by Teng Yue et al.^57^ showed that foliar application of melatonin produced a unique effect of reducing malondialdehyde (MDA) and relative conductivity (REL) in *Solanum nigrum* L. shoots. This study is consistent with the results of Teng Yue’s study, which can restore the osmotic potential of plant cells to a normal state to resist the damage of salt damage to the inside of the plant. Didi Dom Alizet et al.^58^ found that different concentrations of IAA were administered to significantly reduce malondialdehyde (MDA) content at different concentrations compared to Ck. In this study, the free proline content of three desert plants was significantly increased after IAA immersion and application of exogenous melatonin to resist the damage caused by salt damage. Muhammad Ahsan Altaf et al.^59^ showed in a recent study that peppers exposed to CS showed a significant increase in proline levels, while MT application further increased the accumulation of proline in pepper leaves. Xian Xulin et al.^60^ found that exogenous MT treatment significantly enhanced the salinity-alkali tolerance, improved osmoregulation ability, and reduced cell membrane damage of M9-T337 seedlings, and the contents of Pro, SS, SP and St in the leaves of M9-T337 seedlings were further increased after the application of exogenous MT under saline-alkali stress. Xian Xulin’s study was similar to the results of soluble sugars and soluble proteins in the three desert plants in this study, increasing the nutrients in the plants to reduce the effects of saline-alkali soil on the three desert plants. It is worth discussing that malondialdehyde in T1 (12) was greater than that in CK in the paper, and this may have occurred because the saline soil in this experimental design was more damaging to the plants, resulting in an increase in malondialdehyde concentration in the T1 (12) group. Similar results have been reported in the study by Shi Zhenxi et al.^61^ When MT was added under alkali stress, the MDA content of the MT group (100 μmol/L MT) was higher than that of the CK group as determined on the first and second day. Hu Shilian et al.^62^ found that the malondialdehyde content under sodium carbonate stress was greater than that in the CK group when 100 μmol/L MT was added for 3d, 6d, 9d and 12d of stress.

Antioxidant enzymes mainly include superoxide dismutase (SOD), peroxidase (POD), catalase (CAT), and ascorbate peroxidase (APX) in plants, and their primary function is to remove reactive oxygen species from the body to maintain the stability of the enzyme system. MT is a potent antioxidant molecule and an important free radical scavenger that promotes the activity of antioxidant enzymes.^63^ Gull Maria et al.^7^ found that exogenously applied IAA and its precursor L-TRP play an essential role in mitigating the adverse effects of salt stress and that IAA may mitigate the damage caused by salt stress by maintaining endogenous hormone levels and increasing the activity of antioxidant enzymes, which in turn increases the growth rate of potatoes and enhances plant resistance to salt stress conditions. In the study of Ramadan A et al.^64^ both 2 μmol/L and 10 μmol/L IAA could increase the activity of antioxidant enzymes in all parts of tea seedlings under cadmium stress, thereby reducing the effects of cadmium toxicity. Yuxuan Wang et al.^65^ found that the benefits of MT were most significant at a specific concentration (50 μmol⋅L^−1^). This optimal concentration can effectively increase the maximum diameter of peony flowers and enhance the activities of SOD and CAT antioxidant enzymes. This is consistent with the results of this study, which showed that melatonin application increased the antioxidant enzyme activity of three desert plants. Manzer H et al.^66^ found that increased synthesis of Pro and enhanced activities of antioxidant enzymes (SOD, APX, DHAR, MDHAR, and GR) involved in the defense system were observed in seedlings when MT was applied under La toxicity. Ma Qiuxiang et al.^67^ demonstrated the ability of exogenous MT to maintain ROS homeostasis by up-regulating the expression of genes associated with antioxidant enzymes, enhancing their activities, and attenuating lipid peroxidation to maintain ROS homeostasis, thereby delaying aging.

## Conclusion

Saline soils harm the growth and physiology of *Sarcozygium xanthoxylon*, *Nitraria tangutorum*, and *Ammopiptanthus mongolicus*. Exogenous hormone priming is a effective technique for mitigating the effects of saline-alkali soils on these three desert plants. The results showed that the synergistic effect of soaking seeds in exogenous IAA and applying exogenous melatonin had a positive effect on seed germination, growth traits, osmoregulation, chlorophyll a, b, and total chlorophyll, and antioxidant enzyme activities in plants under cadmium stress. Under the synergistic effect of the two hormones, the three desert plants could be treated most appropriately. Principal component and membership function analyses were used to evaluate the most preferred saline-alkali tolerant plants. IAA and melatonin are both endogenous hormones present in plants, and when they are applied externally, they trigger dynamic changes in the content of other hormones in the seed or plant. The future research direction is to screen out which genes are related to the salinity tolerance of plants after the application of IAA and melatonin in the application of IAA and melatonin, and the changes of endogenous hormones and secondary metabolites in seeds or plants after the application of IAA and melatonin. In addition, we should continue to explore and research new plant hormones that can potentially improve plants’ ability to resist salinity.
